# Effects of different HIIT protocols on exercise performance, metabolic adaptation, and fat loss in middle-aged and older adults with overweight

**DOI:** 10.7150/ijms.96073

**Published:** 2024-06-24

**Authors:** Mon-Chien Lee, Yu-Chun Chung, Priskila Cherisca Thenaka, Ya-Wen Wang, Yea-Lih Lin, Nai-Wen Kan

**Affiliations:** 1 Center for General Education, Taipei Medical University, Taipei, Taiwan.; 2 Graduate Institute of Sports Science, National Taiwan Sport University, Taoyuan, Taiwan.; 3 School of Medical Laboratory Science and Biotechnology, College of Medical Science and Technology, Taipei Medical University, New Taipei, Taiwan.; 4 School of Nutrition and Health Sciences, College of Nutrition, Taipei Medical University, Taipei, Taiwan.

**Keywords:** obesity, HIIT, exercise training, blood lipids

## Abstract

**Introduction:** There is evidence that aging and obesity are associated with increased oxidative stress and chronic inflammation. High-intensity interval training (HIIT) may be superior to moderate-intensity continuous training (MICT) in anti-inflammatory and anti-obesity benefits. Therefore, the objective of this study is to determine which HIIT prescriptions will be more effective in reducing fat accumulation, inflammation, and improving metabolic adaptation and exercise performance in middle-aged and older overweight adults.

**Methods:** Thirty-six middle-aged with overweight adults were divided into one of three groups: 1. L-HIIT group: the long-interval HIIT group (4 × 4 min Exercise/4 min Rest), 2. M-HIIT group: the medium-interval HIIT group (8 × 2 min Exercise/2 min Rest), 3. Control group: no exercise training intervention. All groups underwent the training stage for eight weeks (three sessions per week), followed by a detraining stage of four weeks in order to investigate the effects induced by different HIIT interventions on inflammation, metabolic adaptation, anti-fatigue and exercise performance, and fat loss

**Results:** There was a significant physiological response in the change rate of heart rate (HR) after an acute L-HIIT session compared with an acute M-HIIT session (ΔHR: ↑49.66±16.09% vs ↑33.22±14.37%, *p*=0.02); furthermore, systolic blood pressure (SBP) and diastolic blood pressure (DBP) decreased significantly following a single L-HIIT session. After an eight-week training stage, the L-HIIT and M-HIIT groups exhibited a significant increase in aerobic capacity (ΔVO_2peak_), with values of +27.93±16.79% (*p*<0.001) and +18.39±8.12% (*p*<0.001), respectively, in comparison to the control group. Furthermore, in the L-HIIT group, the anaerobic power of relative mean power (RMP) exhibited a significant increase (*p*=0.019). However, following a four-week detraining stage, the adiponectin concentration remained 1.78 times higher in the L-HIIT group than in the control group (*p*=0.033). The results of blood sugar, blood lipids, body composition, and inflammatory markers did not indicate any improved it did not indicate any improvements from the two different HIIT protocols.

**Conclusions:** The results indicate that an eight-week L-HIIT or M-HIIT intervention (three sessions per week, 32 minutes per session) may be an effective approach for improving aerobic capacity. It can be posited that L-HIIT may be a more advantageous mode than M-HIIT for enhancing anaerobic power, adipokine levels, and improving blood pressure in an aged and overweight population due to the induced physiological responses.

## Introduction

Overweightness and obesity are recognized as national epidemics and growing global public health problems, that may be the result of relatively recent changes to lifestyle habits and increased consumption of high-calorie processed foods 1. Furthermore, individuals tend to become less physically active as they age, resulting in a decrease in overall energy expenditure; this has significant implications for energy balance 2. The accumulation of intra-abdominal adipose tissue leads to an increase in the number of senescent cells, as well as a hypoxic microenvironment, which together lead to an increase in the secretion of pro-inflammatory factors 3. Another hallmark of obesity is the presence of chronic low-grade inflammation and sustained oxidative stress (OS). The increased oxidative stress results in the damage of cellular structures, while the insufficient production of antioxidant mechanisms leads to obesity-related complications 4. Thus, obesity has been shown to be significantly associated with diabetes, hypertension, cardiovascular disease, and mortality 5. Additionally, with age, various organ systems and life functions gradually decline. During the aging process, antioxidant components in the body decrease, and the body's ability to scavenge free radicals decreases, resulting in the accumulation of biological macromolecules and structural damage 6. Thus, the gradual and irreversible accumulation of oxidative damage caused by reactive oxygen species (ROS) affects key aspects of the aging process and leads to impaired physiological function, increased disease incidence, and a shortened lifespan 7. We believe that, for middle-aged and elderly people especially high-risk groups such as those with overweight and obesity coping strategies should be explored early to prevent risks. For example, interventions to seek improvement through diet, nutritional supplements, or regular exercise.

Physical exercise is structured physical activity resulting in specific physiological and biochemical responses 8. It is one of the most common non-pharmaceutical methods for preventing non-communicable diseases and improving health status 9. In recent years, high-intensity interval training (HIIT) has become a popular fitness trend due to its time-saving feature and effectiveness 10. Repeated short bursts of intense anaerobic activity (VO_2max_ ≥ 85-90% in healthy subjects, VO_2max_ ≥ 80% in obese and other clinical populations) are interspersed with passive recovery or low-intensity exercise recovery periods, usually of less than 30 minutes 11. A systematic review study has shown that HIIT is a time-saving exercise strategy that further improves waist circumference, body fat percentage, aerobic fitness, and cardiometabolic activity when compared to traditional moderate-intensity sustained aerobic exercise, especially for overweight and obese adults 12. Additionally, 85-90% of overweight/obese women who adhered to three weekly HIIT sessions experienced about a 20% improvement in insulin sensitivity at least 10 weeks 12. Obese individuals also showed greater improvements in body composition after HIIT training compared to traditional endurance training 13.

Multiple studies have confirmed that HIIT has a greater afterburn effect than moderate-intensity continuous training (MICT), a physiological response to intense exercise training and exercise that causes the body to burn more calories and increase insulin sensitivity for hours after rest. It can effectively reduce fat and insulin sensitivity in the body, affect the regulation of cytokines, and maintain a balanced antioxidant status 14, 15. However, exercise prescriptions for HIIT vary in terms of the exercise intensity, cycle period, number of groups, exercise duration, frequency, etc. in addition, oxidative stress and inflammation may be induced during exercise 16. Therefore, it is important to investigate which HIIT protocols are appropriate for overweight or obese middle-aged and elderly individuals to reduce fat accumulation, enhance antioxidant capacity, and decrease inflammation more effectively.

The present study compared the effects of middle and long intervals of HIIT intervention on fatigue, exercise performance, metabolic adaptation, body composition improvements, and physiological response assessment in middle-aged and elderly obese individuals. The aim was to understand the physiological responses, and body composition improvement benefits of different HIIT protocols. We hypothesized that HIIT would improve metabolism and body composition, but that L-HIIT would require a longer duration of exertion than M-HIIT in a single cycle, suggesting that L-HIIT might elicit greater physiological responses and metabolic adaptations.

## Materials and methods

### Participants

We used the Harvard calculator (http://hedwig.mgh.harvard.edu/sample_size/size.html, accessed on 16 December 2021) to determine the sample size, with a significance level of 0.05, a power of 0.8, and a standard deviation of 0.5 for the difference, which means that a total of at least 34 patients must enter the study. For this study, we posted recruitment posters through online social platforms and 36 sedentary participants aged 45-65 years (someone who is about 45 to 65 years old is middle aged) with a body mass index (BMI) between 24 and 35 kg/m^2^ were recruited. Participants who were excluded from the study included those who smoke or drink; have had a stroke; have type 1 or type 2 diabetes; have a neuromuscular disease that prevents them from participating in physical activity; have chronic obstructive pulmonary disease, asthma, interstitial lung disease, or alveolar cystic fibrosis; have metabolic diseases such as thyroid disease, renal disease (kidney or bladder stones), or liver disease; suffer from arrhythmia; wear a heart rhythm regulator; or have severe cardiovascular disease, peripheral vascular disease, or cerebrovascular disease. Participants with epilepsy, rheumatoid arthritis, recent artificial joint implantation or surgery within the past six months, migraine, acute thrombosis, hernia, recent strenuous exercise or muscle soreness within the 24 hours before the experiment, severe food allergies, hospitalization within the past three months, or existing cognitive disabilities, and those taking any drugs that affect the function of the nervous system, could not participate in the experiment. This study was approved and reviewed by the Joint Institutional Review Board of Taipei Medical University (TMU-JIRB, no. N202103041, Taipei, Taiwan) and registered at clinicaltrials.gov under registration number NCT06383442. This study was conducted in accordance with the Declaration of Helsinki. Prior to the experiment, all participants provided written informed consent.

### Experimental Design

**Figure 1:**
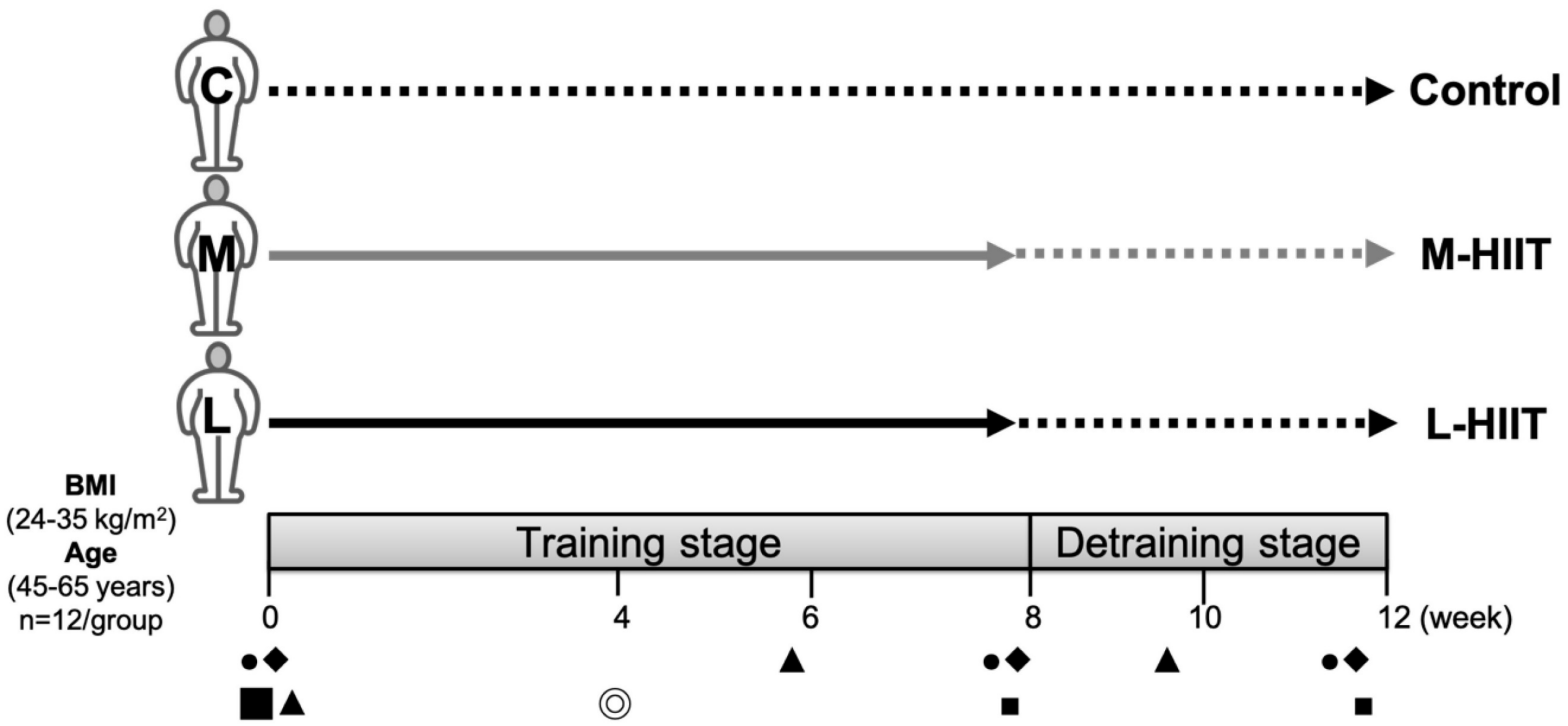
Experimental design.

Participants were evenly distributed according to a counterbalanced design and divided into three different groups by the body fat percentage of subjects: 1. L-HIIT group: the long-interval HIIT group (4 × 4 min Exercise/4 min Rest), 2. M-HIIT group: the medium-interval HIIT group (8 × 2 min Exercise /2 min Rest), 3. Control group. Target intensities of each interval bout, exercise and rest (Ex./R.), were 85-90% VO_2peak_/65-70% VO_2peak_ zones, respectively. The entire exercise duration per session for both the L-HIIT and M-HIIT groups was 32 minutes. Subjects in both HIIT groups performed the L-HIIT or M-HIIT intervention for 8 weeks (3 sessions/week, 24 min/session) during the training stage, followed by a 4-week detraining stage without any exercise. The changes in physiological and biochemical indicators were observed at the pretraining (0^th^ week), training (8^th^ week), and detraining stages (12^th^ week). Subjects were asked to ride a spinning bike for 5 minutes as a warm-up before exercise training. The detailed training protocol is shown in **Table 1**. **Table 2** presents the participants' characteristics. Prior to our intervention, there were no significant differences in age, body composition, or blood pressure between the L-HIIT, M-HIIT, and control groups (*p*>0.05). The experimental design is shown in **Figure 1**. From the beginning to the end of the experiment, none of our subjects dropped out of the experiment.

### Experimental Procedures

All participants underwent body composition and resting blood biochemistry analysis three days before and after the eight-week HIIT intervention. Additionally, a graded exercise load test (GXT) was conducted before formal training, and an acute physiological and biochemical response assessment was conducted during the fourth week of the HIIT intervention. The exercise training and testing were supervised by certified athletic trainers. It is not recommended to engage in training within one hour after a meal, or if subjects have low blood pressure, alcohol intoxication, severe lack of sleep, or other physical discomfort. If the above situation occurred, the participant was asked to postpone or reschedule the start of the exercise training to ensure sufficient recovery. No adverse events occurred to the subjects during the experiment. The training equipment was a stationary spin bike (Cardio Master, MYDO SPORTS, Inc., Xiamen, China), and a heart rate belt (VERITY SENSE, Polar Electro, Inc., Bethpage, NY, USA) was used to monitor the training intensity.

### Body Composition Measurement

All subjects' height, weight, waist circumference, hip circumference, sum of skinfold, and body fat percentage were measured before the intervention (at week 0), after eight weeks of HIIT intervention (at week 8), and after the following four weeks of detraining (at week 12). The sum of skinfold thickness at seven sites (triceps, chest, subscapular muscle, upper ilium, abdomen, front thigh, and leg) was measured using a skinfold caliper (Beta Technology, Santa Cruz, California, USA). Each measurement was taken in duplicate, and, if the difference exceeded 2 mm, it was taken in triplicate. In addition, body fat percentage was measured using the Inbody 570 device (Inbody, Seoul, Korea), based on the bioelectrical impedance principle at multiple frequencies (1, 5, 50, 260, 500, and 1,000 kHz). All subjects were asked to fast for at least 8 h before the test. During the test, subjects stood on the bottom electrode with their arms extended at an angle of 30° to the trunk and held the sensing handle with both hands, without moving or speaking, as previously described 17.

### Blood Biochemistry Index Analysis

In order to understand the subjects' lipid metabolism and post-exercise inflammatory response after exercise, resting blood was collected from all subjects at weeks 0, 8, and 12. Metabolism-related indicators, such as blood glucose (GluAC), triglycerides (TG), low-density lipoprotein (LDL), and high-density lipoprotein (HDL) protein, were analyzed using a fully automatic blood biochemistry analyzer (Hitachi 7060, Hitachi, Tokyo, Japan). Additionally, an automatic analyzer (AU 5820, Beckman Coulter Inc., CA, USA) was used to analyze creatine kinase (CK) and high-sensitivity C-reactive protein (hs-CRP) levels. Furthermore, inflammatory response indicators such as TNF-R1, TNF-R2, and adiponectin were also detected and analyzed using an enzyme-linked immunosorbent assay (ELISA) kits (R & D SYSTEMS, MN USA).

### Diet and Physical Activity Instruction

Participants were instructed to abstain from taking any nutritional supplements or medications during the experiment. They were also advised to avoid engaging in strenuous physical activity for the three days prior to the experiment and to maintain their usual dietary habits as much as possible.

### Aerobic Capacity Test

Before commencing exercise training, a graded exercise test (GXT) was performed using a stationary bike ergometer (ergo_bike Premium 8i, Flugplatzstr, Germany) to assess the subject's VO_2peak_ and corresponding HR_max_. These data served as a baseline for setting the HIIT training intensity. Participants were asked to maintain their daily routines and avoid intense exercise for three days before the GXT. The test was conducted at least 2 hours after a meal and was preceded by baseline measurements of resting heart rate and blood pressure. To ensure safety, the test was postponed if the participant's systolic blood pressure exceeded 160 mmHg, if the diastolic blood pressure exceeded 100 mmHg, or if the heart rate exceeded 100 bpm prior to the test. Immediate termination of the test occurred if the participant experienced severe chest pain, significant difficulty breathing, dizziness, sudden pallor, cyanosis, or abnormal blood pressure (systolic > 200 mmHg, diastolic > 110 mmHg, or > 20 mmHg drop from baseline), or if the participant asked to stop. Heart rate was monitored via a connected heart rate monitor (VERITY SENSE, Polar, Bethpage, NY, USA), and oxygen consumption was analyzed using a gas analyzer (K4b^2^ breath gas analyzer, Milan, Italy). The bike test followed a modified Bruce protocol with a fixed pedaling rate of 60 rpm 18. Starting at 20 Watts for a 3-minute warm-up (stage 0), the power increased by 10 Watts per minute until exhaustion. Exhaustion was determined by criteria such as no increase in oxygen consumption and heart rate with workload, a respiratory exchange ratio (RER) exceeding 1.0, and a Borg's Rate of Perceived Exertion (RPE) exceeding 17 on a 6 to 20 scale. Before and after the 0th, 8th, and 12th weeks of HIIT intervention, a progressive maximal exercise load test was conducted as an assessment of aerobic capacity, evaluating VO_2peak_ as a measure of aerobic capacity.

### Wingate Anaerobic Test (WAnT)

Referring to previous studies 19, all participants underwent an anaerobic power assessment through a 30-second “all-out” test using the classic Wingate Anaerobic Test (WAnT, Monark Exercise AB, Vansbro, Sweden). Following a proper warm-up, the seat height was individually adjusted for each participant, and toe clips were secured to prevent feet from slipping off the pedals. Participants were instructed to exert maximal effort during the sprint. Once the pedal speed reached 120 rpm, an additional resistance equivalent to 7.5% of their body weight was automatically applied to the friction belt of the bicycle. Participants were instructed to maintain a maximal sprint effort for the entire 30-second period.

### Physiological Responses and Muscle Fatigue Assessment to an Acute HIIT Session

During the initial stage of the exercise training, in the fourth week following the HIIT intervention, subjects underwent an evaluation of exercise physiological responses and muscle fatigue before and after a single HIIT session. A Polar heart rate monitor (Polar Accurex, Bethpage, NY, USA) worn on the subject's wrist was employed to detect heartbeats, while an automatic electronic blood pressure monitor (Omega 1400) measured systolic and diastolic blood pressure. Additionally, a handheld lactate meter (Lactate Pro2, ARKRAY, Kyoto, JAPAN) was utilized to collect trace amounts of blood from the subjects' fingertips for analysis. Furthermore, RPE scale (6-20) was employed to assess the overall degree of physical exertion and fatigue experienced by the participants (either involving the entire body or specific parts) during activities. Prior to the experiment, subjects were briefed on the significance of each level on the rating of perceived exertion (RPE) scale to enhance their understanding of the sensory characteristics associated with different exertion levels.

Muscle fatigue assessment involved using a myotonometer (Myoton-Pro, Myoton AS, Tallinn, Estonia) to measure the following parameters at the vastus lateralis and vastus medialis regions: (1) Muscle Tension: The oscillation frequency was calculated using F=fmax, which reflects muscle tension (Frequency); (2) Muscle Hardness: The stiffness of the muscle was determined using a specific formula (Stiffness); (3) Muscle Elasticity: The logarithm of the damping oscillation attenuation was calculated using a specific formula that indicates the elasticity of the muscle (Decrement) and the degree of relaxation (R=tR-t1). These four parameter values effectively identified the overload state and were used to monitor the muscle fatigue state 20.

### Statistical Analysis

Main data are presented as the mean ± SD. Statistical analyses were performed using IBM SPSS Statistics ver. 24.1 (IBM Co., Armonk, NY, USA). A one-way analysis of variance (ANOVA) was used to compare the differences of change rate in various indicators between each group during the training and detraining stages. A repeated measures ANOVA was used to compare intervention differences within-group at different time points. The statistical significance was set at *p*<0.05. With a significance level of 0.05, a power of 0.8, and a standard deviation of 0.5 for the difference, which means that a total of at least 34 subjects must be included in this study. The effect sizes are discussed in terms of negligible (<0.2), small (0.2-0.5), moderate (0.5-0.8), and large (≥) effects.

## Results

### The Impact of Different HIIT Protocols on Body Composition in Middle-Aged and Older Adults

The results are shown in Table 3. There were no significant differences in weight, body fat percentage, subcutaneous fat thickness, waist and hip circumference, or other indicators at the 0th, 8th, and 12th weeks between or within the groups. Furthermore, a detailed analysis was conducted on the impact of different HIIT modes on the change rate of body composition during the training and detraining stages. The results indicated that there were no significant differences in the change rate of various body compositions in the three groups during the eight-week training period (1^st^-8^th^ week) and the subsequent four-week detraining period (9^th^-12^th^ week). However, in terms of the change rate of various body composition indicators (e.g., body weight, body fat percentage, sum of subcutaneous fat thickness, waist and hip circumference), after the eight-week training period, both the L-HIIT and M-HIIT groups exhibited a decline. Following an additional four-week detraining period, the average change in body composition for both the L-HIIT and M-HIIT groups took on an ascending profile, suggesting a slight decrease in body fat after the eight-week training period, followed by a slight increase. In contrast, the control group did not exhibit similar trends (**Table 3**).

### The Impact of Different HIIT Protocols on Aerobic and Anaerobic Capacity in Middle-Aged and Older Adults

Aerobic capacity assessments were conducted on all participants using the GXT method. The Wingate Anaerobic Test was used to analyze anaerobic exercise performance before and after HIIT interventions at the 0^th^ and 8^th^ weeks, and following four weeks of detraining at the 12^th^ week. As shown in **Table 4**, after eight weeks of HIIT training, aerobic capacity assessments using the GXT method revealed a significant increase in the peak oxygen consumption (VO_2peak_) for both the L-HIIT (*p*<0.001, ES=0.785) and M-HIIT groups (*p*<0.001, ES=0.718). The values surpassed the pre-training levels. However, after four weeks of detraining, the VO_2 peak_ for both the L-HIIT and M-HIIT groups significantly decreased (L: *p*=0.01, ES=0.483; M: *p*=0.03, ES=0.400) compared to the 8^th^ week. The VO_2 peak_ of the control group showed no significant changes at the 0^th^, 8^th^, and 12^th^ weeks. Regarding the anaerobic threshold (AT), no significant differences were observed among the three groups at various time points. Furthermore, after eight weeks of HIIT training, the anaerobic power of the L-HIIT group exhibited significant increases in relative mean power (RMP) (*p*=0.019, ES=0.433) compared to pre-training levels. The M-HIIT and control groups did not show significant changes in RPP or RMP to compare between the 0^th^ and 8^th^ week or between the 8^th^ and 12^th^ weeks.

In addition, the effects of different HIIT modes on the change rate of aerobic capacity and anaerobic power during the training stage and detraining stage were further analyzed (**Table 4**). The results showed that, during the eight-week training period (1^st^〜8^th^ week), the ΔVO_2peak_ of the L-HIIT and M-HIIT groups increased by 27.93 ± 16.79% (*p*<0.001, ES=0.535) and 18.39 ± 8.12% (*p*=0.002, ES=0.517), respectively, significantly higher than that of the control group. During the detraining period (8^th^〜12^th^ week), the ΔVO_2peak_ of the two intervention groups decreased by 13.52 ± 10.15% and 13.94 ± 13.34%, to values that were significantly higher than those of the control group (L: *p*=0.025, ES=0.226; M: *p*=0.021, ES=0.193), but there was no significant difference between the L-HIIT and M-HIIT groups (*p*=0.931). As for the change rates of other indexes: ΔAT (%), ΔRPP (%), ΔRPP (%), and ΔRMP (%), there were no significant differences between the two intervention groups during the training period and after the cessation of training.

### The Effect of Different HIIT Protocols on Inflammatory Markers and Adipocyte Hormones in Middle-Aged and Older Adults

**Table 5** shows that there were no significant differences in the change rate between the groups during the training stage (0^th^〜8^th^ week) for CRP, TNF-R_1_, TNF-R_2_, and adiponectin. Although neither mode of eight weeks of HIIT training had a significant effect on the chronic inflammatory markers in this study. During the detraining stage, ΔTNF-R_2_ was significantly higher in the L-HIIT group than in the M-HIIT (*p*<0.001, ES=0.478) and the control (*p*=0.01, ES=0.409) groups. In addition, TNF-R_2_ at the 12^th^ week was significantly lower than that at the 0^th^ week (*p*=0.29, ES=0.238) and 8^th^ week (*p*=0.01, ES=0.511) in the M-HIIT group. According to the results of inflammatory markers, we could not find any benefit from the interventions of two different HIIT during the training stage.

Regarding adipocyte hormones, after eight weeks of HIIT training, the concentration of adiponectin was not significantly different between groups. However, after four weeks of detraining, the adiponectin concentration remained 1.78-fold in the L-HIIT group than in the control group (*p*=0.033, ES=0.157). In addition, adiponectin at the 12^th^ week was significantly higher than that at the 8^th^ week (*p*=0.009, ES=0.337) in the M-HIIT group. The eight-week L-HIIT intervention presented a phenomenon to improve more adiponectin compared to the M-HIIT and control groups. This effect persisted during the subsequent four weeks of detraining.

### The Influence of Different HIIT Training Modes on the Metabolic Parameters in Middle-Aged and Older Individuals with Overweight

**Table 6** shows that there were no significant differences in GluAC, TC, TG, LDL, HDL, SBP, and DBP indicators within groups at the 0^th^, 8^th^, or 12^th^ weeks and between the groups during the training and detraining stages.

### Comparing the Acute Effects of Different HIIT Exercise Sessions on Physiological Responses and Muscle Fatigue in Middle-Aged and Older Individuals with Overweight

In order to investigate the effects of different HIIT training protocols on the physiological responses to exercise in middle-aged and elderly people, this study compared physiological responses between the L-HIIT and M-HIIT groups in the fourth week after HIIT intervention. The results showed that the ΔHR in the L-HIIT group after training immediately was +49.66±16.09%, which was significantly higher than the ΔHR in the M-HIIT group, which was +33.22±14.37% (*p*=0.020, ES=0.223). After exercise, the ΔRPE in both L-HIIT and M-HIIT groups increased by 96.42±44.22% and 71.63±44.49%, respectively, with no significant difference between two groups (*p*=0.185). The blood lactate concentration after a single HIIT session was significantly increased multiple times in both HIIT groups, but the change rate between L-HIIT and M-HIIT groups (+500.68±323.36% vs +328.51±161.00%) were not significantly different (*p*=0.113).

Regarding the post-exercise BP changes, after a single 32-minute session of each of the two HIIT modes, the L-HIIT group exhibited a greater decrease in the SBP and DBP than the M-HIIT groups but there were no significant differences between two groups (ΔSBP: -10.42±12.73% vs -2.92±8.27%, *p*=0.122; ΔDBP: -6.51±6.54% vs -2.44±11.31%, *p*=0.293). However, the SBP and DBP at the post-exercise were significantly lower than those at the pre-exercise (SBP: *p*=0.016, ES=0.267; DBP: *p*=0.006, ES=0.349) only after a single L-HIIT session. The phenomenon of post-exercise hypotension was not observed following a single M-HIIT session. From the physiological responses to a single session of HIIT, despite the consistent intensity and cumulative time of 32 minutes for both L-HIIT and M-HIIT, and equal durations of exercise and recovery periods, the differences in the number of cycles and exercise duration (4 × 4 min Ex./4 min R vs. 8 × 2 min Ex./2 min R) resulted in higher heart rates and noticeable post-exercise hypotensive responses in the L-HIIT group.

As shown in **Table 8**, no significant differences were found between or within the groups for vastus lateralis fatigue-related parameters. In addition, regarding the muscular fatigue responses in the vastus medialis, the muscle elasticity after a single M-HIIT session was 1.02±0.17, which was significantly lower than the muscle elasticity of 1.09±0.13 at pre-training (*p*=0.039. ES=0.200). No significant differences in other muscle fatigue-related parameters (e.g., tone, stiffness, and relaxation) were found between or within the groups. After a single HIIT session, M-HIIT resulted in less muscle elasticity in the vastus medialis compared to L-HIIT. Both groups engaged in an HIIT cycling exercise of the same intensity and duration (32 minutes per session). However, distinct patterns of muscle fatigue were observed in different muscle groups. The M-HIIT group showed higher levels of fatigue in the vastus medialis muscle.

## Discussion

This study revealed that, after eight weeks of HIIT training of different types (Long-interval-HIIT vs. Medium-interval HIIT) and a subsequent detraining period for 4 weeks, while there were no significant changes in body composition, there was a notable enhancement in cardiorespiratory fitness. Moreover, L-HIIT resulted in a more pronounced increase in anaerobic power and showing greater benefits in adiponectin levels compared to M-HIIT.

The fat loss mechanisms of HIIT are thought to involve increases in physical activity and metabolic rate, post-exercise fat oxidation, and post-exercise appetite decrease 21. After HIIT, anaerobic glycolysis is inhibited; however, ATP can still be resynthesized through PCr degradation and a breakdown of triglyceride storage 22. Moreover, it enhances the fatty acid oxidation capacity of both the entire body and skeletal muscles. This could be attributed to the catecholamines generated by HIIT, which facilitate the removal of lactic acid and H^+^, leading to the re-synthesis of glycogen and thereby increasing fat oxidation 21, 23. On the other hand, some studies have also pointed out that HIIT will increase to a greater extent the levels of lipolytic hormones (catecholamines, cortisol, glucagon, and growth hormone) 24. These hormones act on adipose tissue, induce the hydrolysis of triglycerides, and release free fatty acids (FFA), which are transported to active muscle cells and the mitochondrial matrix, where they undergo beta oxidation 25. Elsewhere, an eight-week intervention of HIIT in young men completing a running protocol three times a week (cycle: 4 × 4 min Ex./4 min R, exercise intensity: 90-95% HRmax/70% HRmax) showed a reduction of approximately 1.5 kg (2%) in total body fat 26. A twelve-week HIIT running intervention study, where the rest period was reduced to 3 minutes (4 × 4 min Ex./3 min R) while maintaining the same exercise intensity (90-95% HRmax/70% HRmax) led to a significant decrease in body fat percentage of 7-8% 27. Moreover, another study targeting middle-aged individuals (34-46 years old), which conducted a 12-week HIIT intervention with a protocol that involved six 3 min bouts of high-intensity exercise (90% VO_2peak_) followed by 3 min of low-intensity exercise (50% VO_2peak_), found a significant (11%) improvement in body fat 28. In our study, after eight weeks of L-HIIT and M-HIIT cycling training, the reduction in body fat percentage was approximately 0.77% and 0.23%, respectively, while the control group showed an increase of about 1.55% over the same period. Although the differences were not statistically significant, the lack of significant reduction in body fat may have been influenced by factors such as the duration of HIIT intervention and the mode of exercise (cycling). A study assessing the duration of HIIT found that long-term training (≥12 weeks) has a greater effect on reducing waist circumference compared to short-term HIIT 29. Additionally, long-term training leads to a greater reduction in overall body fat in overweight and obese populations. Therefore, the training duration may influence weight and fat outcomes 30. Previous research has reported that, in a 12-week trial of HIIT three times a week (cycle: 5-7 × 2 min Ex./1 min R; intensity: 95% VO_2peak_/70% VO_2peak_), body mass and fat mass were significantly reduced and VO_2peak_ was significantly increased in adult men 31.

In our study, eight consecutive weeks of L-HIIT and M-HIIT significantly increased VO_2peak_ compared with levels before the intervention. However, there was a notable decrease in VO_2peak_ after a four-week detraining period (**Table 4**). VO_2peak_ is the highest oxygen consumption achieved during an exercise test. It does not necessarily represent maximum oxygen consumption but rather the highest value achieved during the test. It can be used to measure an individual's aerobic capacity during a specific exercise test, especially when testing environment or subject factors prevent reaching VO_2max_ 32 It involves the inhalation of air, diffusion of O_2_ across the pulmonary capillary membrane, binding to hemoglobin in the blood, transportation through the cardiovascular system, and diffusion of oxygen from the blood into muscle cells 33. Finally, it represents the maximum rate at which mitochondrial enzymes utilize oxygen to generate energy-rich substrates for biological functions 34. HIIT has been reported to enhance VO_2peak_ by increasing stroke volume and maximal cardiac output (central adaptation) 35. In addition, HIIT increases the recruitment of rapidly oxidative and glycolytic muscle fibers, which may also increase muscle contractility; changes in muscle contractility promote venous blood return and maintain cardiac output 36. It is suggested that this may be associated with the more pronounced benefits of post-exercise hypotension (PEH) observed in the L-HIIT group (**Table 7**). On the other hand, increased muscle contractility also seems to improve anaerobic capacity. However, in this study, although the anaerobic power performance of the L-HIIT group improved after 8 weeks of intervention compared with before the intervention, it was not significant, and no significant difference was found between the groups. As found by a previous study, it appears that longer and more intense HIIT training programs may lead to significant improvements in anaerobic capacity 37.

Lactate is an intermediate metabolite between glycolic acid enzymatic hydrolysis and mitochondrial respiration. It is formed through the conversion to acetone by cellular LDH and is subsequently removed during glycogenesis and oxidation cycles in tissues. Additionally, lactate can be transformed in the Cori cycle into glucose and hence enhance the availability of energy substrates 38. Previous research has shown that HIIT increases peak blood lactate accumulation, thereby enhancing the availability of energy substrates and helping to transiently improve post-exercise performance. Therefore, trained anaerobic athletes typically have higher lactate levels 39. An earlier study also indicated that trained female cyclists exhibit higher lactate concentrations after HIIT and reach peak levels faster compared to untrained females. Furthermore, the increase in lactate levels during exercise is accompanied by elevated free fatty acid transport and glycerol levels 40. However, in the present study, a single test conducted at the fourth week after the intervention to compare L-HIIT and M-HIIT found that, although the blood lactate concentration increased significantly in both groups compared to pre-test levels. The L-HIIT group showed a higher change rate by 500.68%, but not significant, than that of 328.51% in the M-HIIT group (**Table 7**).

Previous studies have shown that HIIT can markedly improve total fat mass and abdominal fat percentage in older adults 41, and even regulate blood sugar and triglyceride concentrations 42. However, some studies suggest that HIIT may impair exercise performance 43 by inducing lipid peroxidation, protein oxidation, and inflammatory reactions; or affecting the functions of structural proteins and contractile proteins 44. According to previous research on the impact of endurance exercise training on adiponectin levels and its receptors in middle-aged obese individuals, it was found that a 16-week intervention could reduce body fat, enhance VO_2max_, and increase the concentration of adiponectin. The study included two groups: a moderate exercise group, which exercised 5 days per week for 30 minutes per session without a specified heart rate zone, and a high-intensity exercise group, which exercised at 80-90% HR_max_. 45. However, with a 16-week HIIT intervention three times a week (4 × 4 min, 90% HR_max_), there was a trend for improvement in body composition (2.4% decrease in body weight and 2.4% decrease in BMI). In addition, our results showed that TNF-α significantly decreased and adiponectin significantly increased, which contrasts with most research findings that suggest exercise training, accompanied by a reduction in body fat, typically leads to an increase in adiponectin and a decrease in inflammatory responses 46. Research individuals with obesity has found that the key factor in reducing body-related inflammatory markers (including TNF-α, TNF-R1, TNF-R2, IL-6, CRP, etc.) through dietary and exercise interventions (both aerobic and resistance) is achieving a significant decrease in overall body fat 47. White adipose tissue is not only a storage site for energy but also a crucial endocrine organ in the human body. It secretes various adipokines including leptin, adiponectin, resistin, and others. Adiponectin, in particular, plays a role in regulating fat metabolism and influencing blood lipid levels, glucose metabolism, and inflammatory responses 48, 49, 50. In this study, an eight-week L-HIIT exercise program performed three times per week increased adiponectin levels by 22.37% compared to baseline, and this effect was sustained through the twelfth week. This suggests that the duration or frequency of the exercise intervention may be insufficient to effectively reflect improvements in blood lipid status, glucose metabolism, and inflammatory responses. Nevertheless, the relationship between exercise and metabolic adaption remains inconclusive and may be affected by factors such as exercise duration, intensity, age, and others. Consequently, further research is required to elucidate the underlying mechanisms.

There are several limitations to this study. Firstly, during participant recruitment, only the principles of inclusion and exclusion were confirmed, and there was no specific restriction on the gender ratio, leading to an imbalance in each group (Con: 1 male and 11 female; L-HIIT: 3 male and 9 female; M-HIIT: 3 male and 9 female). However, after conducting gender-specific corrections, no significant changes in the data were observed. Secondly, although there are still divergent opinions on the effectiveness of HIIT at improving body composition, most studies have concluded that longer-term training, referring to HIIT of longer duration, is more effective at reducing body fat. Thirdly, in this study, both training protocols L-HIIT and M-HIIT were set to the same exercise/rest intensity (85-90% VO_2peak_/65-70% VO_2peak_), exercise duration per session and exercise frequency. The only difference between groups was the interval mode, with either long-interval HIIT (4 × 4 min Ex./4 min Rest) or medium-interval HIIT (8 × 2 min Ex./2 min Rest). In the future, we would like to compare HIIT of different intensities or increase the training period and intensity.

## Conclusions

In conclusion, this study suggests that an eight-week intervention of long-interval HIIT (4 × 4 min Ex./4 min Rest) or medium-interval HIIT (8 × 2 min Ex./2 min Rest) at least three times per week could improve aerobic capacity. It can be postulated that L-HIIT may be a more advantageous mode than M-HIIT for enhancing anaerobic power, adipokine levels, and improving blood pressure in an aged and population with overweight due to the induced physiological responses.

## Figures and Tables

**Table 1:** Exercise training protocols.

	L-HIIT	M-HIIT	Control
Intensity (Exercise/Rest)	85-90% VO_2peak_/ 65-70% VO_2peak_	85-90% VO_2peak_/ 65-70% VO_2peak_	N/A
Interval Mode	4 × 4 min Ex. /4 min R.	8 × 2 min Ex. /2 min R	N/A
Exercise Duration per Session	32 min	32 min	N/A
Exercise Frequency	3 sessions/week	3 sessions/week	N/A

L-HIIT is the long-interval HIIT group; M-HIIT is the medium-interval HIIT group.

**Table 2:** Participants' basic information.

Parameters	L-HIIT (n=12, 1 male; 11 female)	M-HIIT (n=12, 3 male; 9 female)	Control (n=12, 3 male; 9 female)
Age (years)	48.42 ± 15.48	46.33 ± 12.40	47.58 ± 15.44
Height (cm)	160.53 ± 6.89	164.92 ± 7.31	163.19 ± 8.59
Weight (kg)	69.79 ± 13.99	71.23 ± 10.52	71.95 ± 8.94
BMI (kg·m^-2^)	26.92 ± 3.92	25.88 ± 3.12	26.92 ± 2.42
Body fat (%)	37.21 ± 5.52	34.80 ± 7.24	34.30 ± 5.69
Total skinfold thickness (mm)	215.22 ± 42.77	203.69 ± 34.60	218.49 ± 57.25
Waist circumference (cm)	86.78 ± 13.45	86.44 ± 8.46	88.50 ± 7.99
Hip circumference (cm)	103.84 ± 8.84	98.17 ± 20.22	101.15 ± 5.31
HR _rest_ (bpm)	79.75 ± 7.51	81.64 ± 8.61	74.00 ± 14.34
SBP (mmHg)	117.33 ± 16.00	118.73 ± 12.07	114.08 ± 15.08
DBP (mmHg)	68.67 ± 8.86	73.18 ± 10.07	72.08 ± 12.29

Data are presented as mean ± SD. L-HIIT is the long-interval HIIT group; M-HIIT is the medium-interval HIIT group. BMI, body mass index; HR, heart rate; SBP, systolic blood pressure; DBP, diastolic blood pressure.

**Table 3:** The impact of different HIIT protocols on body composition in middle-aged and older adults.

Parameters	Group	0^ th^ week	8^th^ week	12^th^ week	Training stage (1^st^-8^th^ Week) Change rate (%) Δ (95% CI)	Detraining stage (9^th^-12^th^ Week) Change rate (%) Δ (95% CI)
Body weight (kg)	L-HIIT	69.79±13.99	69.16±15.18	69.73±15.34	-1.19 (-2.99 to 0.61)	0.82 (-0.13 to 1.76)
	M-HIIT	71.23±10.52	70.39±9.59	70.73±9.78	-1.01 (-2.34 to 0.33)	0.46 (-0.32 to 1.23)
	Control	71.95±8.94	71.80±8.94	71.55±8.97	-0.18 (-1.24 to 0.87)	-0.36 (-1.02 to 0.29)
Body fat (%)	L-HIIT	37.21±5.52	37.02±6.26	37.22±6.63	-0.77 (-2.52 to 0.97)	0.59 (-1.61 to 2.79)
	M-HIIT	34.80±7.24	34.77±7.46	35.08±7.33	-0.23 (-1.88 to 1.41)	1.08 (0.92 to 3.12)
	Control	34.30±5.69	34.83±5.85	34.14±5.66	1.55 (-1.28 to 4.38)	-1.80 (-4.61 to 1.00)
Skinfold thickness (mm)	L-HIIT	215.22±42.77	191.48±31.67	206.26±27.70	-10.29 (-15.16 to -5.42)	8.40 (3.41 to 13.40)
	M-HIIT	203.69±34.60	185.21±41.19	193.66±41.48	-9.48 (-16.43 to -2.53	4.74 (0.35 to 9.14)
	Control	218.49±57.25	202.69±51.88	205.71±47.35	-6.66 (-12.19 to -1.13)	2.95 (-5.95 to 11.86)
Waist circumference (cm)	L-HIIT	86.78±13.45	85.58±14.51	86.79±12.61	-1.55 (-3.75 to 0.65)	1.94 (-1.80 to 5.67)
	M-HIIT	86.44±8.46	85.86±8.16	87.84±8.16	-0.54 (-3.60 to 2.52)	2.37 (0.18 to 4.56)
	Control	88.50±7.99	89.40±7.76	88.41±6.32	1.11 (-1.58 to 3.81)	-0.86 (-4.17 to 2.44)
Hip circumference (cm)	L-HIIT	103.84±8.84	102.93±9.54	104.50±10.72	-0.98 (-3.02 to 1.06)	1.64 (-1.74 to 5.01)
	M-HIIT	98.17±20.22	97.89±20.11	98.67±20.44	-0.22 (-2.18 to 1.74)	0.70 (-0.77 to 2.16)
	Control	101.15±5.31	102.47±4.82	102.44±5.73	1.35 (-0.19 to 2.89)	-0.02 (-2.13 to 2.09)

Data are presented as mean ± SD. L-HIIT is the long-interval HIIT group; M-HIIT is the medium-interval HIIT group.

**Table 4:** The impact of different HIIT protocols on the aerobic and anaerobic exercise capacities in middle-aged and older adults.

Parameter	Group	0^th^ week	8^th^ week	12^th^ week	Training stage (1^st^-8^th^ Week) Change rate (%) Δ (95% CI)	Detraining stage (9^th^-12^th^ Week) Change rate (%) Δ (95% CI)
VO_2peak_ (mL·kg^-1^·min^-1^)	L-HIIT	21.98±7.00	27.57±7.24^**^	23.67±6.14^#^	27.93 (17.27 to 38.59)^b^	-13.52 (-19.97 to -7.07)^b^
	M-HIIT	21.98±3.49	26.03±4.53^**^	22.12±3.68^#^	18.39 (13.24 to 23.55)^b^	-13.94 (-22.42 to 5.47) ^b^
	Control	27.18±6.43	26.36±6.73	25.55±6.14	-2.60 (-10.62 to 5.41)^a^	-2.26 (-9.62 to 5.10)^a^
AT (mL·kg^-1^·min^-1^)	L-HIIT	11.42±4.31	12.67±3.06	12.23±2.54	26.03 (-7.63 to 59.70)	0.50 (-14.74 to 15.73)
	M-HIIT	12.69±4.11	13.29±3.56	11.23±3.69	9.99 (-7.58 to 27.56)	-13.73 (-29.01 to 1.54)
	Control	13.59±3.65	15.02±5.68	13.83±6.08	14.04 (-12.54 to 40.61)	-3.49 (-23.10 to 16.12)
RPP (Watt·kg^-1^)	L-HIIT	4.40±1.44	4.32±1.22	4.26±1.30	14.46 (1.92 to 26.99)	0.01 (-7.99 to 8.00)
	M-HIIT	4.28±1.19	4.65±0.72	5.15±1.09	17.43 (-9.75 to 44.61)	11.52 (-0.56 to 23.61)
	Control	4.93±1.62	5.30±1.48	5.30±1.45	10.42 (0.88 to 19.95)	2.19 (-13.82 to 18.20)
RMP (Watt·kg^-1^)	L-HIIT	2.76±1.01	3.20±0.97^*^	3.07±0.78	20.02 (5.33 to 34.71)	-1.75 (-9.79 to 6.29)
	M-HIIT	2.86±0.78	3.20±0.61	3.52±0.88^&^	18.80 (-4.18 to 41.79)	10.58 (-1.16 to 22.31)
	Control	3.51±0.95	3.68±0.83	3.93±1.21	7.94 (0.69 to 15.20)	7.28 (-10.25 to 24.81)

Data are presented as mean ± SD. L-HIIT is the long-interval HIIT group; M-HIIT is the medium-interval HIIT group. AT, Anaerobic Threshold (mL·kg^-1^·min^-1^); RPP, Relative Peak Power; RMP, Relative Mean Power. Within-group comparisons between week 0 and week 8, * represents a significant difference (*p*<0.05); ** represents a significant difference (*p*<0.01); within-group comparisons between week 8 and week 12, # represents a significant difference (*p*<0.05); within-group comparisons between week 0 and week 12, & represents a significant difference (*p*<0.05), respectively; and a difference in the superscripted numbers (a, b) represents a significant difference between groups (*p* <0.05).

**Table 5:** The effect of different HIIT protocols on inflammatory markers and adipocyte hormones in middle-aged individuals.

Parameter	Group	0^th^ week	8^th^ week	12^th^ week	Training stage (0^th^-8^th^ Week) Change rate (%) Δ (95% CI)	Detraining stage (9^th^-12^th^ Week) Change rate (%) Δ (95% CI)
CRP (μg·dL^-1^)	L-HIIT	0.27±0.40	0.16±0.14	0.15±0.15	29.55 (-64.60 to 123.70)	53.63 (241.66 to 69.76)
	M-HIIT	0.21±0.16	0.20±0.20	0.19±0.17	10.53 (-46.88 to 67.94)	8.13 (-21.33 37.58)
	Control	0.21±0.19	0.15±0.10	0.24±0.25	-5.1875 (-39.28 to 28.91)	86.22 (-8.62 107.27)
TNF-R_1_ (pg·mL^-1^)	L-HIIT	794±359	761±299	841±346	-3.13 (-14.30 8.05)	11.54 (-4.79 27.88)
	M-HIIT	814±268	811±221	790±278	-0.14 (-9.11 8.83)	-3.48 (-12.07 5.10)
	Control	738±191	816±177	798±146	9.81 (0.23 19.39)	-0.16 (-11.41 to 11.09
TNF-R_2_ (pg·mL^-1^)	L-HIIT	3809±1304	3686±1240	4092±1426	-1.98 (-14.32 to 10.36)	12.11 (-0.38 to 24.60)^b^
	M-HIIT	4361±1564	3923±900	3319±944^#&^	-3.07 (-21.23 to 15.08)	-15.56 (-22.58 to -8.53)^a^
	Control	3983±1483	4221±1348	3498±1,149	11.11 (-6.31 to 28.53)	-15.96 (-26.57 to -5.34)^a^
Adiponectin (pg·mL^-1^)	L-HIIT	3925±1793	4585±2548	4,818±2983^b^	22.37 (-3.91 to 48.65)	0.75 (-11.92 to 13.42)
	M-HIIT	3064±1045	2697±836	3304±1429^#ab^	-7.03 (-23.63 to 9.56)	17.85 (7.87 to 27.84)
	Control	3583±2159	3999±3320	2,705±1061^a^	7.73 (-18.32 to 33.78)	-28.91 (-18.05 to 10.93)

Data are presented as mean ± SD. L-HIIT is the long-interval HIIT group; M-HIIT is the medium-interval HIIT group; CRP, C-reactive protein; TNF, tumor necrosis factor. Within-group comparisons between week 0 and week 8, * represents a significant difference (*p*<0.05); within-group comparisons between week 8 and week 12, # represents a significant difference (*p*<0.05); within-group comparisons between week 0 and week 12, & represents a significant difference (*p*<0.05), respectively; and a difference in the superscripted numbers (a, b) represents a significant difference between groups (*p* <0.05).

**Table 6:** The impact of different HIIT training modes on metabolic parameters in middle-aged individuals.

Parameter	Group	0^th^ week	8^th^ week	12^th^ week
GluAC (mg·dL^-1^)	L-HIIT	94.25±9.12	95.33±7.89	91.25±14.19
	M-HIIT	95.25±17.78	98±12.46	95.17±16.21
	Control	95.33±14.42	94.08±15.32	94.58±16.84
TC (mg·dL^-1^)	L-HIIT	189.17±28.39	191.83±46.02	190.25±38.75
	M-HIIT	181.08±35.62	181.83±33.36	190.42±40.02
	Control	189.08±26.57	178.25±24.42	194.92±29.00
TG (mg·dL^-1^)	L-HIIT	115.92±49.52	135.33±215.40	112.75±121.70
	M-HIIT	147.25±99.89	115.92±49.52	130.75±66.83
	Control	141.67±99.89	141.67±59.99	110.08±31.27
LDL (mg·dL^-1^)	L-HIIT	111.00±28.72	110.00±33.44	112.75±33.23
	M-HIIT	118.08±33.97	122.17±32.88	124.67±35.95
	Control	119.17±21.87	113.17±23.25	130.50±28.61
HDL (mg·dL^-1^)	L-HIIT	62.08±14.82	68.83±16.11	67.00±15.66
	M-HIIT	48.50±10.50	51.75±9.50	53.00±12.23
	Control	53.17±10.52	54.00±10.97	57.50±14.15
SBP (mmHg)	L-HIIT	117.33±16.00	122.00±18.13	123.92±18.83
	M-HIIT	118.73±12.07	120.73±19.20	121.55±13.61
	Control	114.08±15.08	117.75±15.82	119.42±14.78
DBP (mmHg)	L-HIIT	68.67±8.86	78.00±6.89	79.33±6.85
	M-HIIT	73.18±10.07	83.27±10.08	83.64±10.06
	Control	72.08±12.29	75.00±11.96	77.75±11.12

Data are presented as mean ± SD. L-HIIT is the long-interval HIIT group; M-HIIT is the medium-interval HIIT group; Glu, glucose, TC, total cholesterol; TG, triglyceride, HDL, high-density lipoprotein; LDL, low-density lipoprotein; SBP, systolic blood pressure; DBP, diastolic blood pressure.

**Table 7:** The impact of different HIIT training modes on physiological parameters in an acute HIIT session.

Parameter	Group	Pre-	Post-	Change rate (%) Δ (95%CI)
HR (bpm)	L-HIIT	77.75±8.00	115.58±9.60^**^	49.66 (39.44 to 59.88)^b^
	M-HIIT	80.42±8.34	108.17±10.14^**^	35.12 (27.49 to 42.74)^a^
RPE	L-HIIT	7.25±1.48	13.75±1.71^**^	96.42 (68.32 to 124.51)
	M-HIIT	7.92±1.56	13.00±1.28^**^	71.63 (43.36 to 99.90)
Lactate (mmol·L^-1^)	L-HIIT	1.93±0.53	10.44±3.82^**^	500.68 (295.23 to 706.14)
	M-HIIT	2.47±1.00	10.21±4.39^**^	328.51 (226.21 to 430.80)
SBP (mmHg)	L-HIIT	126.42±18.65	116.00±14.12^*^	-7.58 (-13.38 to -1.79)
	M-HIIT	118.33±14.54	115.42±14.51	-2.30 (-6.61 to 2.01)
DBP (mmHg)	L-HIIT	85.67±8.94	80.00±8.92^*^	-6.51 (-10.66 to -2.36)
	M-HIIT	81.17±11.88	78.33±7.64	-2.44 (-9.63 to 4.74)

Data are presented as mean ± SD. HR, heart rate; RPE, rating of perceived exertion; DBP, diastolic blood pressure; SBP, systolic blood pressure. Within-group comparisons between pre and post, * represents a significant difference (*p*<0.05); ** represents a significant difference (*p*<0.01); values in the same column with different superscript letters (a, b) differ significantly, *p* < 0.05.

**Table 8:** Comparison of muscular fatigue responses of the Vastus muscles between different HIIT modes in an acute HIIT session

Parameter	Group	Pre	Post	Change rate (%) Δ(95%CI)	Pre	Post	Change rate (%) Δ (95%CI)
Muscle		Vastus Lateralis			Vastus Medialis		
Tone (Hz)	L-HIIT	20.04±3.38	19.30±3.20	-3.38 (-7.74 to 0.97)	18.38±3.55	18.43±3.16	0.69 (-3.19 to 4.57)
	M-HIIT	21.73±5.01	21.85±5.14	0.60 (-4.14 to 5.34)	18.03±2.99	17.68±3.33	-1.74 (-7.54 to 4.07)
Stiffness (N·m^-1^)	L-HIIT	493±143	464±114	-4.41 (-10.11 to 1.30)	416±114	416±104	2.53 (-3.42 to 8.48)
	M-HIIT	562±183	522±183	-1.55 (-7.21 to 4.10)	392±105	384±129	-3.17 (-14.94 to 8.61)
Elasticity	L-HIIT	1.22±0.21	1.20±0.17	-0.52 (-7.69 to 6.67)	1.10±0.16	1.05±0.14	-3.16 (-11.10 to 4.77)
	M-HIIT	1.21±0.24	1.21±0.19	1.90 (-7.40 to 11.19)	1.09±0.13	1.02±0.17*	-6.51 (-12.79 to -2.35)
Relaxation (ms)	L-HIIT	11.50±3.41	11.84±2.61	5.27 (-2.70 to 13.24)	13.18±3.12	12.88±2.87	-1.46 (-7.17 to 4.25)
	M-HIIT	10.30±3.38	10.23±3.36	-0.16 (-6.55 to 6.24)	14.21±4.39	14.76±5.11	3.67 (-5.66 to 12.99)

Data are presented as mean ± SD. Within-group comparisons between pre and post, * represents a significant difference (*p*<0.05).
